# The effects of locus coeruleus optogenetic stimulation on global spatiotemporal patterns in rats

**DOI:** 10.1162/imag_a_00314

**Published:** 2024-10-15

**Authors:** Nmachi Anumba, Michael A. Kelberman, Wenju Pan, Alexia Marriott, Xiaodi Zhang, Nan Xu, David Weinshenker, Shella Keilholz

**Affiliations:** Department of Biomedical Engineering, Georgia Institute of Technology and Emory University, Atlanta, GA, United States; Department of Human Genetics, Emory University, Atlanta, GA, United States; Molecular Cellular and Developmental Biology Department, University of Colorado Boulder, Boulder, CO, United States

**Keywords:** locus coeruleus, optogenetic-fMRI, complex principal component analysis, quasi-periodic patterns, global signal, resting state

## Abstract

Whole-brain intrinsic activity as detected by resting-state fMRI can besummarized by three primary spatiotemporal patterns. These patterns have beenshown to change with different brain states, especially arousal. Thenoradrenergic locus coeruleus (LC) is a key node in arousal circuits and hasextensive projections throughout the brain, giving it neuromodulatory influenceover the coordinated activity of structurally separated regions. In this study,we used optogenetic-fMRI in rats to investigate the impact of LC stimulation onthe global signal and three primary spatiotemporal patterns. We report small,spatially specific changes in global signal distribution as a result of tonic LCstimulation, as well as regional changes in spatiotemporal patterns of activityat 5 Hz tonic and 15 Hz phasic stimulation. We also found that LC stimulationhad little to no effect on the spatiotemporal patterns detected by complexprincipal component analysis. We hypothesize that localized effects could be dueto engagement of LC modules that support behaviors induced by our specificstimulation parameters, in addition to noradrenergic receptor profiledistributions. Nonetheless, these results show that the effects of LC activityon the BOLD signal in rats may be small and regionally concentrated, as opposedto widespread and globally acting, further supporting emerging evidence of amodular LC.

## 1 Introduction

Resting-state functional MRI (rs-fMRI) is increasingly used to non-invasively studydynamic brain activity and the intrinsic forms of large-scale communication acrossthe brain. Of particular interest are recent studies that have shown a majority ofspatially structured signals captured using rs-fMRI can be summarized or representedby three primary spatiotemporal patterns, also known as quasiperiodic patterns orQPPs (Bolt et al., 2022;Majeed et al., 2011;Yousefi et al., 2018;Yousefi &Keilholz, 2021). The most prominent of these patterns, QPP1, captures thespatiotemporal evolution of the global blood oxygenated level dependent (BOLD)signal. The global signal, typically measured to be the average of all brainactivity over the course of a scan, is often used as a nuisance regressor to reducethe effects of widespread noise in rs-fMRI data (Birn et al., 2006;Parkes et al.,2018;Satterthwaite et al., 2012;Yan et al., 2013); although theexistence of resting-state networks and large-scale patterns of activity have causedmany to question the removal of the global signal from fMRI datasets (Ciric et al., 2017;Fox et al., 2009;T. T.Liu, Nalci, et al., 2017;Murphy &Fox, 2017;Saad et al., 2012). Thesecond strongest spatiotemporal pattern, QPP2, comprised semi-regular waves ofalternating activity between different structures and networks that have beenobserved across species. In humans, QPP2 captures a cyclical pattern ofanticorrelation between the default mode network (DMN) and task positive network(TPN), along with propagation across the cortex during the transition betweenactivation of the two networks (Majeed et al.,2011). In rodents, it manifests as anticorrelation between the rodent DMNand lateral cortical network, the latter of which is believed to exhibit similaractivity to the human TPN (Belloy et al.,2018). The anticorrelated QPP also contributes substantially tofunctional connectivity across the whole brain (Abbas, Belloy, et al., 2019;Belloy etal., 2018;Yousefi et al., 2018).Of note is that most prior studies employed global signal regression, making QPP2the primary spatiotemporal pattern that was observed. A third spatiotemporal pattern(QPP3) is also sometimes evaluated by regressing out QPP2 and extracting the nextstrongest resulting dynamic pattern. QPP3 has been shown to primarily involvebilateral cortical regions and exhibit lateral-medial propagation; however, thefunctional significance of QPP3 is not well understood (Bolt et al., 2022;Meyer-Baese et al., 2024a).

Despite most often being measured during resting state, both of these spatiotemporalpatterns exhibit notable changes during arousal and in correspondence withvigilance-related measures. Specifically, the global signal (also captured by QPP1)is inversely correlated with various measures of vigilance and arousal acrossdifferent species. In humans, an EEG measurement of vigilance was found to beinversely correlated with the amplitude of the global signal (Wong et al., 2013,^2016^). A negative correlation to the global signal was also found with alocal field potential measure of arousal in non-human primates (Chang et al., 2016). A separate study in mice used pupildiameter as a proxy for arousal and showed that changes in pupil diameter wereanticorrelated to changes in global hemodynamics (Pisauro et al., 2016). Additionally, the introduction of caffeine as ameasure of vigilance has been shown to increase anti-correlation between the DMN andthe TPN (Wong et al., 2012), and a wave-likepropagation of phase shifts was reported to occur as a result of infra-slowarousal-related activity (Raut et al., 2021).These relationships suggest that the global signal and QPP1 typically increase instrength as arousal diminishes.

QPP2 also has ties to attention and arousal. Early work showed that time-varyinganticorrelation between the DMN and TPN was related to intra- and inter-individualvariability in reaction time on a psychomotor vigilance task (Thompson et al., 2013). Further analysis showed thatthese fluctuations in reaction time were related to the phase of QPP2 at task onset(Abbas et al., 2016). The contribution ofQPP2 to rs-fMRI is reduced in patients with ADHD compared to healthy controls (Abbas, Bassil, et al., 2019). Taken together,these findings suggest that the strength of QPP2 should increase with increasingarousal. Recent analysis of data from the Human Connectome Project indicates thatQPP1 increases at the expense of QPP2 over the course of a scan, consistent withgrowing drowsiness and prior work that examined QPP1 and QPP2 separately (Meyer-Baese et al., 2024b).

The brainstem noradrenergic locus coeruleus (LC) is a key node in the brain’sarousal system and regulates sleep, stress responses, mood, and cognition (Aston-Jones & Bloom, 1981;Aston-Jones & Cohen, 2005;Benarroch, 2018;Poe et al., 2020;Rajkowski et al., 1994). In recent years, the LC-norepinephrine (LC-NE)system has been recognized as an important contributor to the generation ofspatiotemporal dynamic signals. Of interest to fMRI in particular, it has also beenshown that LC-NE vascular innervation helps to optimize neurovascular coupling,leading to a potential increase in sensitivity of the BOLD signal (Bekar et al., 2012). As the primary source of central NEand potential for brain-wide release, the vast projections of the LC have potentialto influence whole-brain levels of activity. In fact, evidence shows that theactivity of brainstem nuclei may play a significant role in large-scalespatiotemporal signals (van den Brink et al.,2019).Turchi et al. (2018)showedthat inhibition of the basal forebrain decreased the amplitude of global signalfluctuations. However, these effects were more pronounced during less alertbehavioral states, indicating a connection of this effect to arousal and potentiallythe activity of other brainstem nuclei. The origin of QPP2 has been speculated toarise from the activity of neuromodulatory brainstem nuclei (Abbas, Belloy, et al., 2019), and preliminary work hasshown that extreme modulation of NE levels (either up or down) dramatically reducesthe detection of this QPP (Abbas et al.,2018). The LC-NE system has also been hypothesized to coordinate andintegrate activity across structurally segregated regions (Shine, 2019). LC activation has further been shown toaffect large-scale measures of activity by strengthening brain-wide connectivity andeven modulating nodes of the rodent DMN (Oyarzabalet al., 2021;Zerbi et al.,2019).

In this study, we aimed to further investigate the contribution of the LC-NE systemto the composition of the global signal, QPP1 and QPP2 through the utilization ofoptogenetic-fMRI. Optogenetics is optimal for this purpose because, beyond generalcell-type specific stimulation, it has the capacity to employ specific stimulationrates, patterns, and timing. This is critical for the LC because it demonstratescontrasting firing rates and patterns that are associated with distinct arousal andcognitive states. LC neurons have low tonic pacemaker activity (~1–2 Hz)during general wakefulness, high tonic activity (~5–10 Hz) in response tostress, and phasic activity (~15 Hz bursts followed by inhibition) during focusedattention and goal-directed behavior (Aston-Jones& Bloom, 1981;Aston-Jones& Cohen, 2005;Berridge &Waterhouse, 2003;Carter et al.,2010;McCall et al., 2015;Prokopiou et al., 2022;Valentino & Foote, 1988). Thus, using combinedoptogenetic-fMRI with mCherry expressing animals serving as controls, weinvestigated distinct characteristics of the global signal, QPP1 and QPP2 during lowtonic (2 Hz), high tonic (5 Hz), and phasic (15 Hz bursts) stimulation of the LC. Wereport small, spatially-specific changes in global signal distribution under tonicLC stimulation, as well as regional changes in QPP2 involvement during 5 Hz tonicand 15 Hz phasic stimulation in ChR2-expressing animals compared to mCherrycontrols. We also show little to no effect of LC stimulation on these spatiotemporalpatterns as detected by complex principal component analysis (CPCA), an analysisthat simultaneously detects the global signal and the anticorrelated QPP from BOLDsignals (Bolt et al., 2022). These resultsindicate that the neuromodulatory effects as a result of LC optogenetic stimulationon large-scale spatiotemporal patterns are relatively small in magnitude andconcentrated in particular brain regions.

## 2 Materials and Methods

An overview of the methods involved in the optogenetic-fMRI experimentation frominitial surgery to data analysis can be seen inFigure1.

**Fig. 1.**
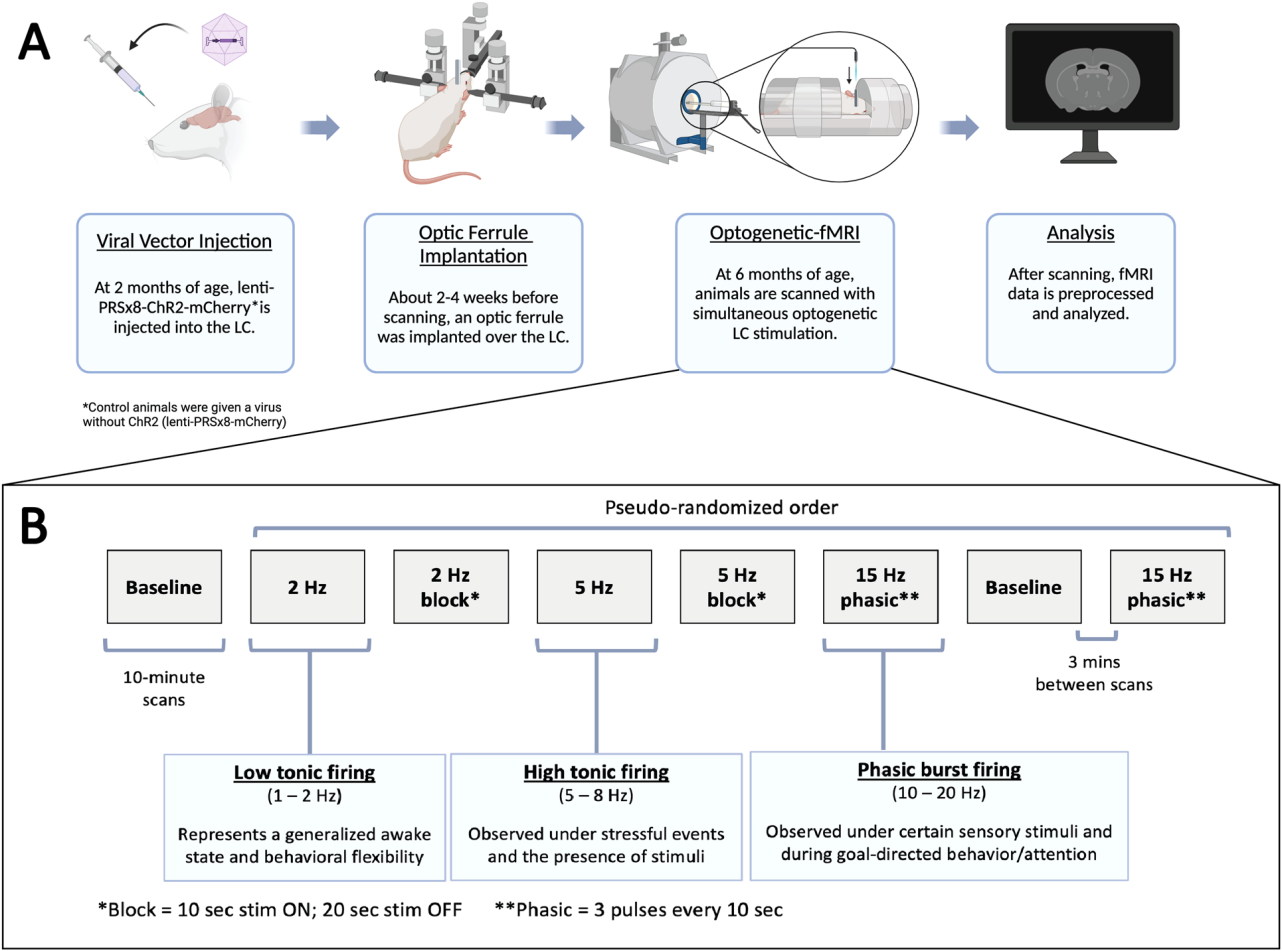
Optogenetic-fMRI experimental methods. (A) Experimental timeline. 2-month-oldrats received bilateral injections of a lentivirus containing the syntheticnoradrenergic promoter PRSx8, the mCherry fluorescent protein, andchannelrhodopsin-2 (ChR2) if they were not control animals. Two to 4 weeksbefore scanning took place, animals were implanted unilaterally with anoptic ferrule over the LC. At 6 months of age, animals underwent an fMRIscanning session with varying levels of simultaneous optogenetic LCstimulation. After scanning, all fMRI data were preprocessed and analyzed.(B) Scanning session timeline. All scanning sessions consisted of eight10-minute fMRI scans with 3 minutes between scans. The LC was stimulatedusing 2 Hz, 5 Hz, and 15 Hz phasic bursts to replicate the common modes offiring displayed by the LC. Each session started with a baseline scan (nostimulation) that was followed by the remaining seven scans in apseudo-randomized order. The block stimulation scans were not used in thisstudy. Created in part withBioRender.com.

### 2.1 Animal preparation

Two cohorts of 6-month-old male and female wildtype Fischer rats were used inthis study. All rats were bred in-house, kept on a 12 hour light/dark cycle(lights on at 7:00 am), and provided access to food and water*adlibitum*throughout the experimental period. Rats were group-housedin groups of two to three animals up until implantation of the optic ferrule,after which they were singly housed until they underwent scanning in the MRImachine (approximately 2-4 weeks). Experimental groups comprised animals of bothsexes. For a breakdown of the sex makeup of each group, refer to (Supplementary Table 1).For information regarding number of subjects and scans, seeTable 1. All protocols used in this studywere approved by the Institutional Animal Care and Use Committee (IACUC) atEmory University, and all procedures were performed in accordance with the IACUCprotocols. All surgeries and experiments were carried out under isofluraneanesthesia, and all efforts were made to minimize the stress and suffering ofthe animals.

**Table 1.** Animal group sizes.

	mCherry Animals (Scans)	ChR2 Animals (Scans)
Baseline	9 (15)	7 (13)
2 Hz	8 (8)	4 (4)
5 Hz	7 (7)	5 (5)
15 Hz phasic	8 (12)	6 (10)

### 2.2 Surgery and pupillometry

Stereotaxic surgery was performed on all rats at approximately 2 months of age.Rats were initially put under anesthesia with 5% isoflurane. Isoflurane was thenlowered to 2% for the remainder of the surgery, and rats were given ketoprofen(5 mg/kg, s.c.) directly following incision. To avoid contact with the sagittalsinus a 15-degree head tilt was utilized and bilateral injections (1.3µl/hemisphere; AP: -3.8 mm from lambda, ML: +/- 1.2 mm, DV: 7.0 mmfrom skull surface) of a lentivirus under control of the synthetic noradrenergicpromoter, PRSx8, were used to express either ChR2-mCherry or mCherry selectivelyin the LC (Abbott et al., 2009;Hwang et al., 2001;Vazey & Aston-Jones, 2014). After theinfusion, the needle was left for 5 minutes after which it was moved 1 mmdorsally and 2 minutes were allotted to permit appropriate viral diffusion atthe injection site. Viruses were allowed to express for approximately 4 monthsprior to fMRI scanning.

Approximately 2–4 weeks before undergoing MRI scanning, rats wereimplanted with a custom fiber optic ferrule (FG200UCC, 200 um, 0.22 NA, 7.5 mmlength; Thorlabs) above the LC to enable delivery of optogenetic stimulation.The hemisphere in which the LC was targeted was randomized for each rat so thatstimulation of both the left and right LC was incorporated into group analyses.First, the skull was implanted with three MRI-compatible screws to hold theferrule in place. The aforementioned surgical protocol was then performed withan optic ferrule being implanted dorsal to the LC (AP: -3.8 mm from lambda, ML:+/- 1.2 mm, DV: 6.5–6.8 mm from skull surface).

**Fig. 2.**
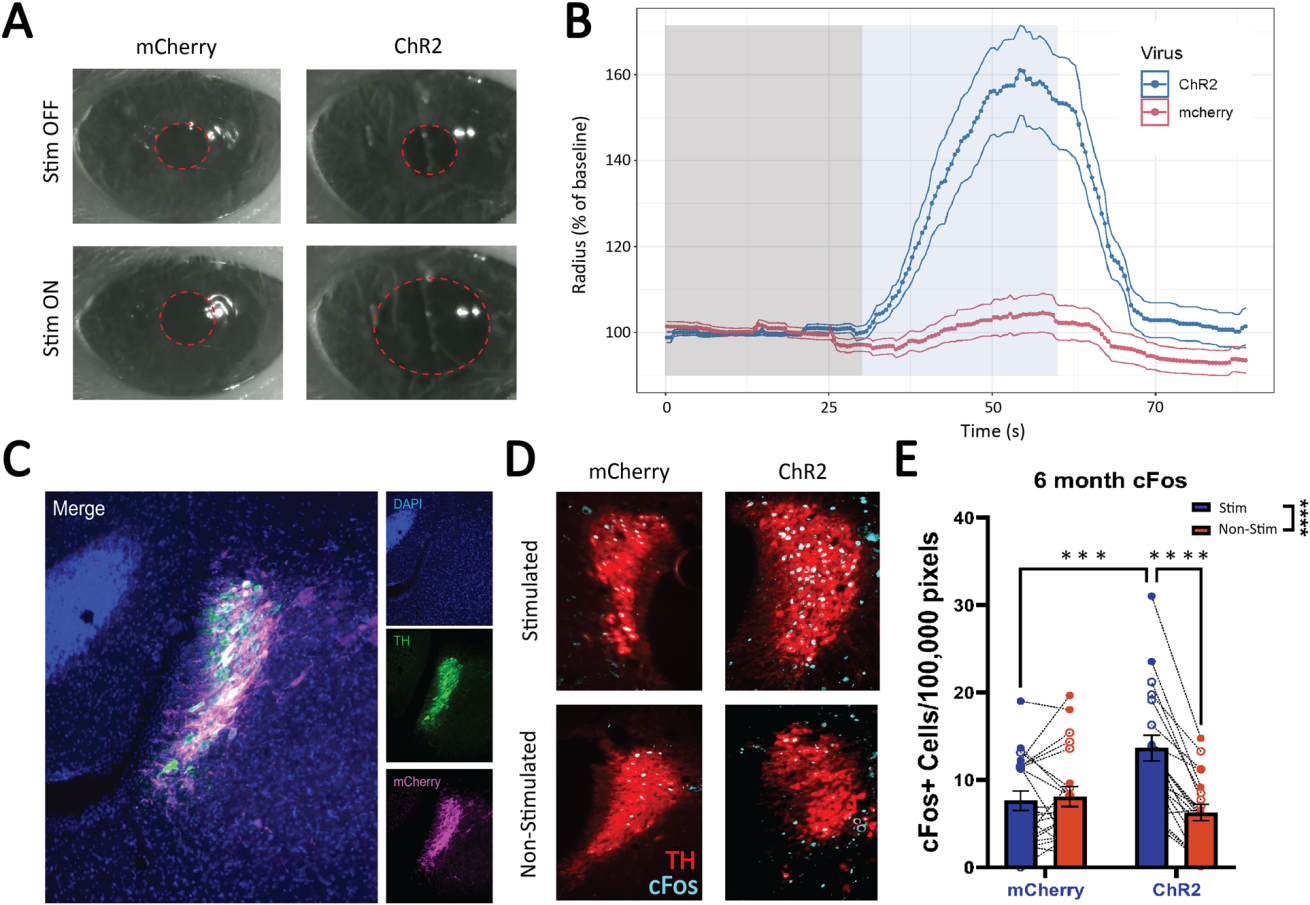
Confirmation of LC optogenetic stimulation via pupillometry andimmunohistochemistry. Viral expression and response to optogeneticstimulation in the LC was verified using a combination of pupillometryand immunohistochemistry. (A) Visually notable increases in pupildiameter experienced during LC stimulation via optic fiber. (B) Thechange in pupil diameter was quantified and compared between mCherry andChR2 animals using an open source pupillometry toolkit (Privitera et al., 2020). (C) Confirmation ofviral expression with robust fluorescence of mCherry (pseudo-coloredmagenta) limited to the LC (marked by TH; green (D) c-Fos expression wasused to confirm the activation of LC neurons roughly 90 minutes after 5Hz constant optogenetic stimulation. Shown is a representative image ofan mCherry and a ChR2 animal and the differences in c-Fos expression(teal) between the stimulated and non-stimulated hemispheres of the LC(TH; red). (E) c-Fos counts were quantified for both the mCherry andChR2 animals at 6 months.

Stimulation of the LC results in pupil dilation (Y. Liu, Rodenkirch, et al., 2017;Zerbi et al., 2019), providing a useful measure to verify successfulviral expression and optic ferrule targeting. We performed this verification viapupillometry following previously published protocols (Privitera et al., 2020). Our setup involved the useof a Raspberry Pi NoIR Camera Module V2 night vision camera, an infrared lightsource, and a Raspberry Pi 4 Model B (CanaKit). During optic ferruleimplantation, 90 seconds of pupil recordings were taken of the eye ipsilateralto the hemisphere of the stimulated LC. Black tape was used to block light fromthe stimulation that may have impacted pupil dilation. The recordings comprised30 seconds of baseline (no stimulation) followed by 30 seconds of LCstimulation, followed by 30 seconds of post-stimulation baseline footage (Fig. 2B). Pupil dilation was quantifiedoffline using a previously established pipeline (Privitera et al., 2020). In the event that ChR2-mCherry did notdisplay pupil dilation in response to LC stimulation, the other hemisphere wasimplanted and checked for pupil dilation. If there was still no pupil dilation,animals were re-categorized to the control group. During offline analysis, therewere two animals where pupil recording lengths differed. These videos weremanually checked by an experimenter blinded to experimental condition for thepresence or lack of pupil dilation in response to LC stimulation. Dental cementwas used to secure the implant to the skull, and animals were allowed to recoverfor approximately 2–4 weeks.

### 2.3 fMRI data acquisition

On the day of MRI scanning, rats were initially induced with 5% isoflurane for 5minutes in combination with a 2:3 ratio of O_2_and medical grade air.After initial induction with isoflurane, animals were maintained under2–3% isoflurane for the remainder of handling and cradle preparations.Rats were intubated and administered the paralytic pancuronium dibromide (1.5mg/kg/hour, s.c.; Selleck) immediately after intubation. Through the trachealtube, breathing of the rats was maintained at 1.6 Hz using a ventilator. Animalswere then placed in a custom-made MRI cradle where a custom-madetransmit-receiver surface coil was placed over the brain and an optic fiberpatch cord was connected to the optic ferrule. Animals were then head-fixed withear bars and their teeth secured in a bite bar to limit head motion duringscanning. To prevent the escape of light during stimulation, a black marker wasused to ‘blackout’ the headcaps and tape was placed over theconnection between the optic fiber and the optic ferrule implant. Eye ointmentwas placed over both eyes prior to scanning, and isoflurane was reduced to 1.3%and maintained at this level for the rest of the scanning session. Heart rateand blood oxygen levels of the animals were monitored throughout the scanningsession using a pulse oximeter that was connected to the hindpaw of the animal.Temperature of the rat during scanning was maintained at 37 ±0.5°C using a heated water bath system.

All fMRI scans were acquired using a 9.4T Bruker Biospec MRI scanner with a 20 cmhorizontal bore. Scans were 10 minutes in length and were gradient-echoecho-planar imaging (EPI) with partial Fourier encoding with a factor of 1.4.The following parameters were used to acquire the images: isotropic voxel size= 0.5 mm^3^, matrix size = 70 x 70, slice number =24, TE = 15 ms, TR = 1250 ms, flip angle = 90°, andbandwidth = 216.45 kHz. All scans were phase-locked so that eachacquisition was set to occur at a multiple of the animal’s respiratoryrate. In this study, the repetition time of 1250 ms (0.8 Hz) was employed toacquire images at the frequency of every other breath of the 1.6 Hz respirationrate. This practice was adopted to ensure that each image slice was obtained atthe same point in the animal’s respiratory cycle and therefore limitmotion-induced artifacts as a result of chest cavity movements duringrespiration (Pan et al., 2020). Asingle-volume reversed blip EPI image with the same parameters was acquiredbefore each 10-minute scan and used for topup correction (Andersson et al., 2003;Smith et al., 2004). Saturation bands were used withall functional EPI scans to minimize signal from outside of the brain, and eachscan was preceded by 10 dummy scans for system calibration purposes.

Four different levels of LC stimulation were incorporated into this study:baseline (i.e., no stimulation), 2 Hz, 5 Hz, and a 15 Hz phasic stimulation(comprising 3 pulses of light at 15 Hz every 10 seconds). These frequencies werechosen to mimic the distinct firing modes that have been observed to occurnaturally in the LC: low tonic, high tonic, and phasic burst firing (Aston-Jones & Bloom, 1981;Aston-Jones & Cohen, 2005;Berridge & Waterhouse, 2003;Carter et al., 2010;McCall et al., 2015;Prokopiou et al., 2022;Valentino& Foote, 1988). Each scanning session consisted of a total ofeight scans that were presented in a pseudo-randomized order: two baseline scansand two scans each at 2 Hz, 5 Hz, and 15 Hz phasic stimulation (Fig. 1). For the scans in which 2 Hz and 5 Hzstimulation was presented, one of the two scans was acquired with constantstimulation at that frequency for the entirety of the 10 minutes, while a blockdesign of stimulation (10 seconds of stimulation and 20 seconds of rest) wasemployed for the other scan. The block stimulation scans were not analyzed inthe current study and will not be mentioned further. Each session started with abaseline scan, a 5 Hz constant scan was employed as the fourth scan and alwaysfollowed by the second baseline scan; the placement of all other stimulationlevels was randomized for each session. Placement of the 5 Hz constantstimulation scan was maintained to ensure optimal expression of c-Fos duringstaining approximately 90 minutes later (seeSection 2.5) (Tillage et al.,2020,^2021^). All optogeneticstimulation was done with 60 mW of laser power and 10 ms-long pulses. Threeminutes were allotted between scans to allow for norepinephrine levels to returnto baseline post-stimulation.

### 2.4 Preprocessing

Preprocessing of fMRI data was done using the*Rodent Whole-Brain fMRIData Processing Toolbox*(Xu,Zhang, et al., 2023). Through use of this toolbox, the followingpreprocessing steps were performed: slice time correction, motion correction,topup correction, nuisance regression (constant, linear, and quadratic trends aswell as six motion regressors), normalization, bandpass filtering(0.01–0.1 Hz), spatial smoothing (FWHM = 0.5 mm), and registrationto and seed extraction from the functional SIGMA-Wistar atlas (Barrière et al., 2019).

### 2.5 Tissue preparation and immunohistochemistry

After scanning was complete, the rats were removed from the ventilator andperfused immediately with 0.1 M kPBS followed by 4% paraformaldehyde. Afterbrains were extracted, they were placed in 4% paraformaldehyde and storedovernight before being moved to 30% sucrose for a minimum of 3 days beforeslicing. Brains were sliced at 30 um at the level of the LC. Sections wereeither mounted on Colorfrost® Plus slides (Erie Scientific) to becounterstained with neutral red to check for optic ferrule placement (M. A. Kelberman et al., 2023) or stored incryoprotectant until fluorescence staining.

Free floating sections were washed 3 x 5 minutes in 1 x PBS and then incubatedfor 1 hour in blocking buffer (5% normal goat serum, 3% bovine serum albumin in0.1% PBST). Sections were then incubated in chicken anti-tyrosine hydroxylase(TH) (1:1000; abcam ab76442) and rabbit anti-c-Fos (1:3000; abcam ab190289) for48 hours at 4°C. Sections were then washed 3 x 5 minutes in 1 x PBS andincubated for 2 hours with the corresponding secondary antibodies goatanti-rabbit 488 (1:500; Fisher Scientific A11008) and goat anti-chicken 633(1:500; Thermo Fisher Scientific A-21103). Then, sections were washed again 3 x5 minutes and mounted on slides, dried, and coverslipped. A separated set of LCsections were stained using the same protocols for TH and rabbit anti-DsRed(1:1000; Fisher Scientific NC9580775), along with the appropriate secondary goatanti-chicken 488 (1:500; abcam ab150169) and goat anti-rabbit 568 (1:500; ThermoFisher Scientific A-11011) antibodies. These sections were used to visuallyconfirm viral expression within the LC.

A Leica DM6000B epifluorescent upright microscope was used to image the sectionsstained for c-Fos at 10x. Using ImageJ, TH was used as a marker to manuallyoutline the LC. A common Otsu threshold was set, and despeckling was performed.Then, the number of c-Fos labelled cells was counted using standard criteria(pixel size: 100-infinity and circularity: 0.7-1.0). A two-way repeated-measuresANOVA was used to assess differences in c-Fos expression across groupsconsidering virus (ChR2-mcherry and mCherry alone) and hemisphere (stimulatedand non-stimulated) as factors.

### 2.6 Data analysis

#### 2.6.1 Spatial distribution of the global signal

In order to investigate how LC stimulation affects the spatial aspect of theglobal signal, the regional distribution of the global signal was assessed.The global signal was calculated by extracting all timecourses within thebrain region and averaging them into a single timecourse. The global signalsfor each scan were then z-scored by subtracting the mean and dividing by thestandard deviation. The scans and global signals for each group wereconcatenated, and the timecourse of each voxel within the brain wascorrelated to the global signal via a Pearson’s linear correlation.The maps of correlation values of each voxel to the global signal aredisplayed by group. For this analysis, we examined the relative correlationwith the global signal, which results in a spatial map, rather than QPP1,which results in a spatiotemporal pattern, to facilitate comparison to priorstudies of the global signal. For significance testing involving thecingulate cortex, the correlation values for the three parcellations thatcomprised the cingulate cortex in the SIGMA-Wistar functional atlas(Cingulate Cortex 1, Cingulate Cortex 2, and Cingulate Cortex 3) wereaveraged to represent the activity of the cingulate cortex and a two-wayANOVA was performed considering virus (ChR2-mcherry and mCherry alone) andstimulation level (baseline, 2 Hz, 5 Hz, and 15 Hz phasic) as factors. Allanalysis was performed in MATLAB using custom scripts.

#### 2.6.2 QPP analysis

QPP2 was obtained using a repeating pattern detecting algorithm that wasinitially reported in (Majeed et al.,2011) and later modified in (Yousefi et al., 2018). This algorithm starts by taking atemplate, comprising a number of volumes within the scan that is dictated bythe user-defined window length, and uses a sliding window approach tocorrelate this template to each timepoint of the acquired scan. A thresholdis set for the correlation timecourse and at all points at which thecorrelation between the template and the scan is higher than the threshold,the volumes are taken and averaged to create a new template. This process isrepeated until the template does not change after two iterations (cc> 0.9999). The global signal was regressed from each scan prior toapplying the algorithm to ensure detection of QPP2 for comparison with priorstudies (Yousefi et al., 2018). Forgroup QPP analysis, the algorithm was run on the concatenation of all scanswithin the said group.

To assess the frequency of QPP2 occurrence for each group, a histogram of thefinal sliding template correlation (STC) timecourse for each group wascreated. STC values above the standard threshold of 0.2 were considered tobe QPP events. To evaluate the pattern of the QPP2 itself, the finaltemplate provided by the algorithm was observed and compared between groups.To evaluate statistical significance, the percentage of STC values above the0.2 threshold and a measure of cingulate cortex activity in the QPP wereassessed. For the latter, the activity of the cingulate cortex (defined asthe average of the three cingulate cortex parcellations defined by theSIGMA-Wistar functional atlas) during periods of detected QPP activity wasaveraged for each scan and the difference between the maximum and minimumlevels of activity was measured. In both instances, a two-way ANOVA wasperformed considering virus (ChR2-mcherry and mCherry alone) and stimulationlevel (baseline, 2 Hz, 5 Hz, and 15 Hz phasic) as factors. All QPP analyseswere performed in MATLAB using custom scripts. A similar version of thescripts used in this study is available in (Xu, Yousefi, et al., 2023).

#### 2.6.3 Complex principal component analysis (CPCA)

To further investigate the incidence of these spatiotemporal patterns, weperformed complex principal component analysis (CPCA). CPCA is able tosimultaneously detect multiple spatiotemporal structures within BOLDsignals, including QPP1 and QPP2 (Bolt etal., 2022). Specifically, CPCA, applied to complexHilbert-transformed BOLD timecourses through PCA, assesses both themagnitude and phase-lag differences among brain structures. This analysistransforms BOLD signals into real (magnitude) and imaginary (phase)components, with the principal components retaining these attributes. Weconducted CPCA using a Python script that was reported in (Bolt et al., 2022) and is available athttps://github.com/tsb46/complex_pca. Custom MATLAB scripts werethen used to evaluate the incidence of the first three principal components.Incidence of a principal component at any given timepoint was defined bywhich of the first three components explained the most variance in the scanat that timepoint. A two-way ANOVA (performed in MATLAB) was used to assessdifferences in principal component incidence across groups considering virus(ChR2-mcherry and mCherry alone) and stimulation level as factors.

## 3 Results

### 3.1 Multimodal confirmation of optogenetic LC stimulation

Taking advantage of the link between LC activity and pupil dilation, we usedpupillometry to confirm accurate placement of the optic ferrule implant andexpression of the virus (Fig. 2A). Weobserved robust dilation of the ipsilateral pupil in ChR2 expressing animalsduring optogenetic stimulation, while stimulation-driven pupil dilation was notobserved in animals expressing the mCherry control virus. Pupil dilation wasquantified and compared between all mCherry and ChR2 animals (Privitera et al., 2020) (Fig. 2B). Post-mortem staining for mCherry confirmed robustexpression of the virus within the LC (Fig.2C). Further supporting LC activation in response to optogeneticstimulation, staining for the immediate early gene c-Fos revealed strongactivation of the stimulated hemisphere only in the ChR2-expressing animals(Fig. 2D). A two-way repeated-measuresANOVA revealed no main effect of virus (F_(1,39)_= 1.83, p= 0.18), but there was a main effect of hemisphere (F_(1,39)_= 29.03, p < 0.0001), subject (F_(39, 39)_= 5.83,p < 0.0001), and a virus x hemisphere interaction (F_(1,39)_= 37.51, p < 0.0001). Tukey’s post-hoc tests revealedsignificantly elevated c-Fos in the stimulated hemisphere of ChR2 animalscompared to those expressing mCherry alone (p = 0.0006), and there was asimilar outcome when comparing the stimulated to non-stimulated hemispherewithin ChR2 expressing animals (p < 0.0001) (Fig. 2E).

### 3.2 Spatial distribution of the global signal

**Fig. 3.**
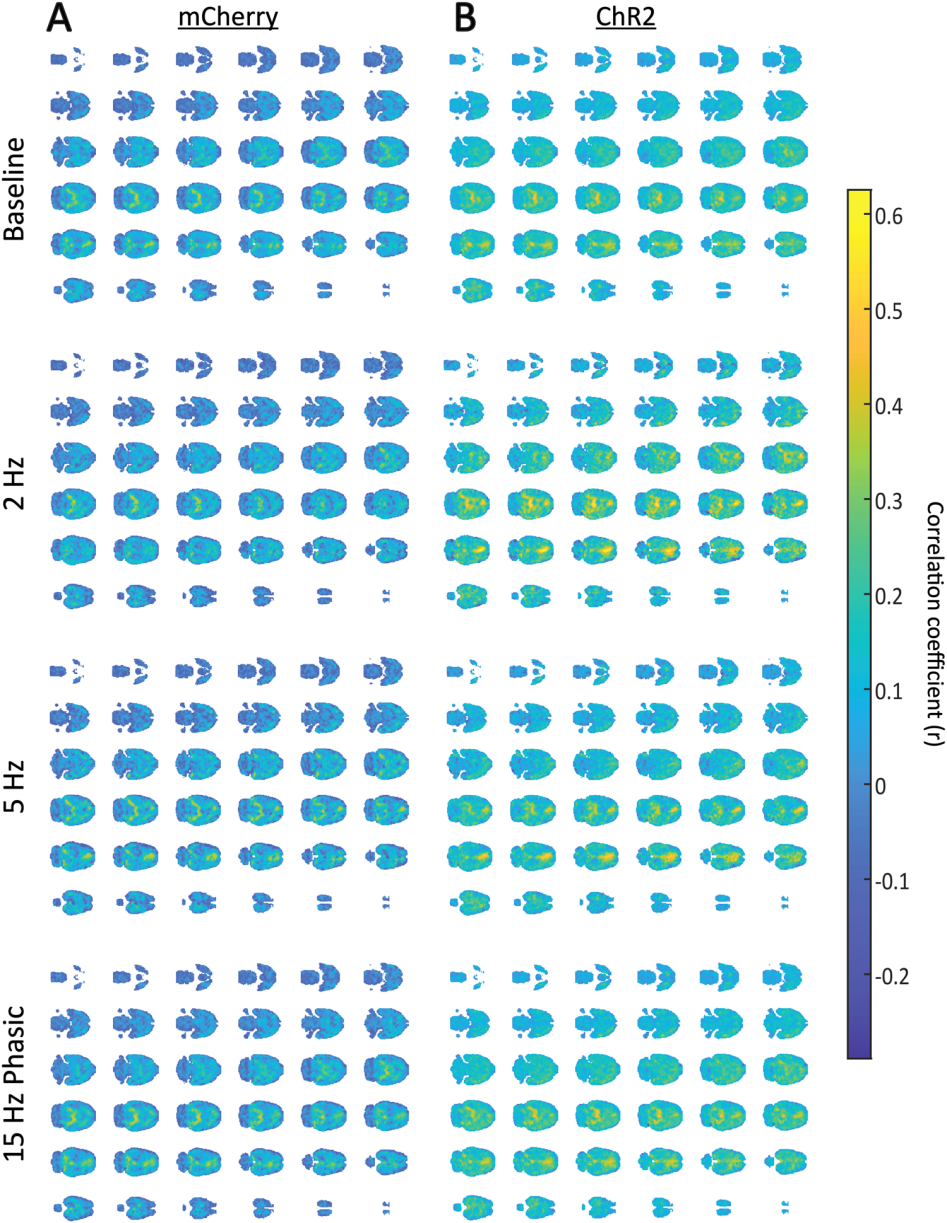
Spatial distribution of the global signal during LC stimulation. (A) Thespatial distribution of the global signal in the mCherry control animalsat different levels of LC stimulation. Correlation is shown inPearson’s correlation coefficients (r). The highest correlationto the global signal was found along the midline in anterior corticalstructures as well as bilaterally in posterior parts of the brain,replicating previous results (Anumba etal., 2023). (B) The spatial distribution of the global signalin ChR2 stimulated animals at different levels of LC stimulation. Thesame pattern of highly correlated structures as seen in the controlanimals was observed here, although with higher correlation seenthroughout the whole brain. When compared to baseline, correlation inthe bilateral posterior region was stronger during 2 Hz LC stimulationand correlation in the anterior medial structure was stronger during 2Hz and 5 Hz stimulation of the LC.

To determine whether LC stimulation affects the level of contribution ofdifferent brain regions to the global signal, we assessed the spatialdistribution of the global signal via a voxel-wise correlation analysis (Fig. 3). In the mCherry control group acrossall levels of stimulation, higher correlation to the global signal was seenalong the midline and bilaterally in posterior regions of the brain (Fig. 3A). This finding replicates previouswork that investigated the spatial distribution of the global signal in anoise-controlled cohort of rats (Anumba et al.,2023). Interestingly, a separate study that looked at co-activationpatterns of the global signal in awake rats found heavier contribution ofsubcortical structures and the prefrontal cortex to the global signal (Ma et al., 2020). This discrepancy may bepartially explained by the use of anesthesia in our current study. While thesame general pattern was observed in the ChR2 stimulated rats as that seen inthe mCherry rats, the correlation values were widely elevated throughout thebrain, including during baseline scans (Fig.3B). Notably, this increase was disproportionately higher in thecentral anterior region, which likely represents the cingulate cortex (Anumba et al., 2023), during 2 Hz and 5 Hztonic stimulation of the LC. Given that this was observed to be the spatialregion that underwent the greatest amount of change, a two-way ANOVA wasperformed on the correlation between the activity of the cingulate cortex andthe global signal. While there were no main effects of stimulation pattern oncingulate cortex contribution to the global signal (F_(3,66)_=0.1779, p = 0.9110), there was a main effect of virus (F_(1,66)_= 4.826, p = 0.0316). In general, animals expressing ChR2 in theLC had stronger correlation between the activity of the cingulate cortex and theglobal signal. There was no additional effect based on the interaction betweenvirus and stimulation pattern on cingulate cortex contribution to the globalsignal (F_(3,66)_= 0.8210, p = 0.4869).

### 3.3 QPP2 analysis

**Fig. 4.**
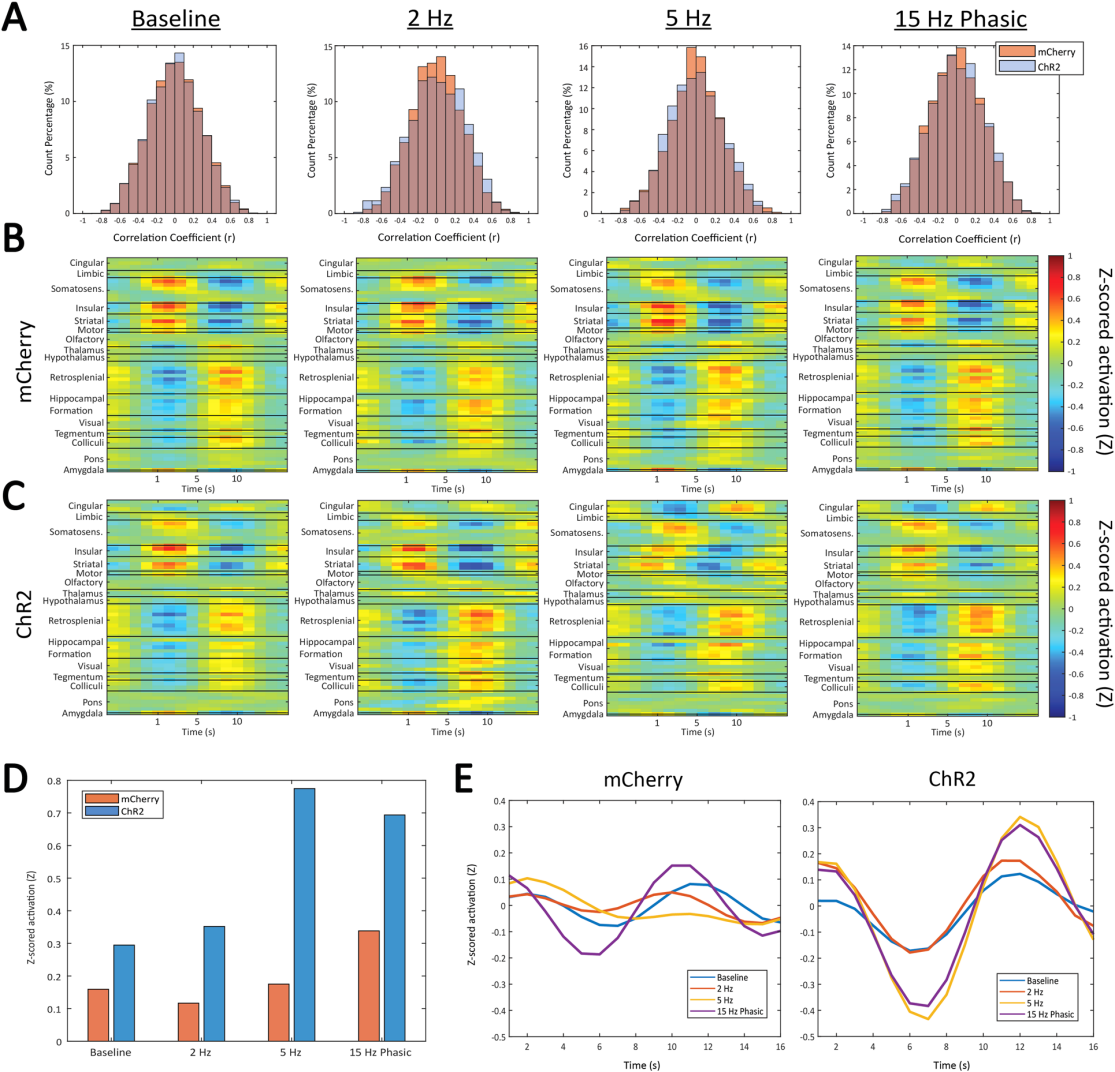
QPP detection and templates. (A) STC histograms for mCherry (orange) andChR2 (blue) animals where values over 0.2 were considered QPP events.Very little difference was shown between the two animal groups; however,a slightly higher percentage of counts were above the 0.2 threshold forthe ChR2 animals during 2 Hz and 5 Hz stimulation. (B) QPP templates, orregional activity within the QPP, for mCherry animals at eachstimulation level. (C) QPP templates, or regional activity within theQPP, for ChR2 animals at each stimulation level. Some minor differencesin regional activity were seen during 5 Hz tonic and 15 Hz phasic LCstimulation. (D) Difference between maximum and minimum activityexhibited by the cingulate cortex in the QPP template. (E) Activity ofthe cingulate cortex during the QPP template across stimulation levelsfor mCherry and ChR2 animals.

A pattern finding algorithm was used to detect the occurrence of QPP2 in thisdataset (seeSection 2for details). TheSTC values of the final QPP template with the scans of each group are shownplotted as a histogram (Fig. 4A). Thethreshold for a QPP event was set to 0.2, and any timepoints at whichcorrelation was above this threshold were considered QPP events. Thedistributions of correlation values for all scan groups showed a near completeoverlap between the mCherry and ChR2 animals at baseline. However, for thedistributions during the 2 Hz and 5 Hz stimulation levels, a higher percentageof correlation values above the absolute value of the 0.2 threshold wereobserved in the ChR2 stimulated animals than in the mCherry controls. Thisindicates that more QPP events were detected in the ChR2 animals during 2 Hz and5 Hz stimulation when compared to the mCherry controls. During 15 Hz phasicstimulation, there was more overlap between mCherry and ChR2 animals above thethreshold, although this overlap was not as complete as it is for the baselinescans. To evaluate these trends, a two-way ANOVA was performed on the percentageof STC values above the 0.2 threshold. However, we found no effect ofstimulation pattern (F_(3,66)_= 0.7457, p = 0.5287),virus (F_(1,66)_= 1.933, p = 0.1691), or a stimulationpattern x virus interaction (F_(3,66)_= 0.962, p =0.416) on the percentage of STC values above the 0.2 threshold.

The QPP template generated for each scan group was also compared across groups toassess the involved activity of each brain region within the QPP (Fig. 4B-C). The templates show the waves ofactivation and deactivation that were exhibited by distinct regions and thatcharacterize the QPP. Notably, certain regions exhibited activity that was inphase with each other and distinctly out of phase with other regions, whereasother regions showed very little oscillatory activity in the pattern at all. Thesomatosensory, insular, striatal, motor, and amygdala regions all exhibitedsimilar activity that was almost exactly anticorrelated to the activity of theretrosplenial cortex, hippocampal formation, and certain deep brain structures.Other cortical regions, such as the olfactory network and cingulate cortex, andsub-cortical regions, such as the thalamus, hypothalamus, and the pons,contributed very little to the dynamic pattern. Importantly, the cingulatecortex showed a much stronger involvement in the QPP of ChR2 animals under 5 Hztonic and 15 Hz phasic LC stimulation compared to any of the control animals orat other stimulation levels in the same group. This cingulate activity, whenpresent, moved in phase with the retrosplenial and hippocampal regions. Furtherfocusing on this region as exhibiting the greatest change, we next isolated theactivity of the cingulate cortex to look at the strength of its involvementduring the QPP, where strength is defined as the difference between its highestand lowest levels of activity in the template (Fig. 4D-E). Overall, the cingulate cortex showed stronger activityin ChR2 animals when compared to mCherry animals, where this increase inactivity is larger during 5 Hz and 15 Hz phasic stimulation. An importantdistinction to be made is thatFigure 4D-Edisplays the activity of the cingulate cortex in the final QPP template, not atthe individual level. It is this template that is correlated across theconcatenated scans of a group to identify QPP occurrences (i.e., wherecorrelation to the template is greater than 0.2). Therefore, to look on anindividual level, the concatenated scans were divided by individual and the QPPoccurrences were averaged for that scan (seeSection 2.6.2for more details). When these individual instances ofQPP activity were assessed, a two-way ANOVA revealed no effect of stimulationpattern (F_(3,66)_= 0.2616, p = 0.8528), virus(F_(1,66)_= 0.08774, p = 0.7680), or a stimulationpattern x virus interaction (F_(3,66)_= 0.2569, p =0.8562) on this measure of cingulate involvement in the QPP. Additionally, wealso observed an apparent phase shift in the activity of the insular andstriatal regions when compared to that of the somatosensory network during 5 Hzstimulation in the ChR2 animals.

### 3.4 Complex principal component analysis (CPCA)

Using the unique feature of CPCA to evaluate regional differences in time-lagactivity, we performed this analysis as a complementary way to observe thespatiotemporal dynamics in the dataset. Previous work in humans revealed thefirst three principal components of CPCA as common spatiotemporal patterns ofactivity often observed in the brain (Bolt etal., 2022). Whether detected with the QPP algorithm or CPCA, thefirst component represents the BOLD global signal, and the second componentcaptures the observed anti-correlation between the DMN and TPN. For this reason,CPCA results in this study were confined to the first three principal components(Meyer-Baese et al., 2024b). In theinterest of continuity, in this section we will refer to the first threeprincipal components as QPP1, QPP2, and QPP3, to remain consistent with thenomenclature used for the QPP algorithm.

**Fig. 5.**
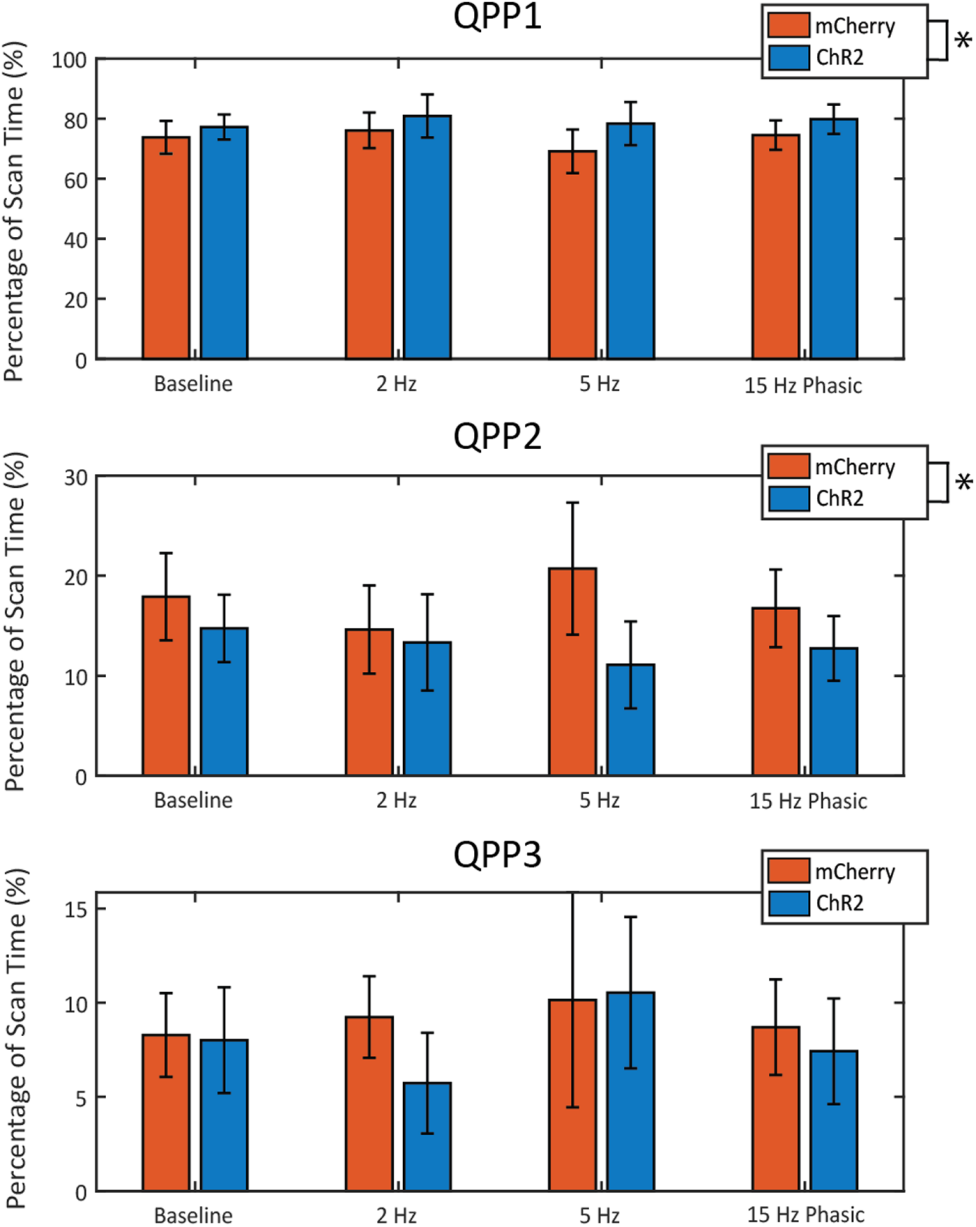
Incidence percentage of the first three QPPs during LC stimulation. Thepercentage of scan time for which each of the first three QPPs wasdominant is displayed in this figure. The differences between thecontrol animals (orange) and the stimulated animals (blue) were comparedfor each level of LC stimulation. Incidence for QPP1 across all groupswas much higher than it was for QPP2 and QPP3. A two-way ANOVA revealedsignificance on account of virus for the first two components; however,post-hoc analysis showed no further significance at the level ofstimulation. Error bars represent the mean +/- SEM.

The effects of LC stimulation on the incidence of each QPP were analyzed bycomparing the percentage of scan time that was dominated by each component forthe mCherry control group and the ChR2 stimulation group (Fig. 5). As expected, across groups and stimulation levels,QPP1 was responsible for a majority of the variance explained (~70%). Incidenceof QPP1 was slightly higher for all ChR2 animals as indicated by a two-way ANOVAthat reported no effect of stimulation level (F_(3,66)_= 0.68,p = 0.5691), but a significant effect of the virus (F_(1,66)_= 6.19, p = 0.0154). There was no additional effect on QPP1incidence based on the interaction between virus and stimulation pattern(F_(3,66)_= 0.29, p = 0.8329). Generally, animalsexpressing ChR2, regardless of stimulation pattern, spent a higher proportion oftime in QPP1 compared to mCherry-expressing animals. The overall incidence ofQPP2 was much lower than was observed for QPP1; however, incidence of QPP2 wasconsistently lower in ChR2 animals when compared to mCherry control animals. Atwo-way ANOVA revealed no effect of stimulation level (F_(3,66)_= 0.39, p = 0.7578), but there was a significant effect of thevirus (F_(1,66)_= 6.42, p = 0.0137). There was noadditional effect on QPP2 incidence based on the interaction between virus andstimulation pattern (F_(3,66)_= 0.8, p = 0.501).Regardless of stimulation level, ChR2-expressing animals generally spent a lowerproportion of time in QPP2 compared to mCherry animals. The overall incidence ofQPP3 was the lowest of the three components, with no consistent trend observedacross stimulation level or virus. A two-way ANOVA revealed no significanteffect of stimulation level (F_(3,66)_= 0.82, p =0.4892) or virus (F_(1,66)_= 0.89, p = 0.3486). Thetiming and incidence of each component during the scan was also assessed at theindividual level; however, no trend was observed as a result of LC stimulation(Supplementary Fig.S3).

## 4 Discussion

Both the BOLD global signal and the anticorrelation between DMN and TPN areassociated with arousal and measures of vigilance (Pisauro et al., 2016;Wong et al.,2012,^2013^,^2016^). The LC plays critical role in arousal, exertingits neuromodulatory influence throughout the brain (Aston-Jones & Cohen, 2005;Berridge & Waterhouse, 2003;Poeet al., 2020). Through the use of optogenetic-fMRI, we have shown thatstimulation of the LC at different frequencies results in relatively small changesin correlation to global signal that can be significant in some regions of thebrain, and that ChR2-expressing rats exhibit higher ratios of QPP1 to QPP2 thanmCherry control rats.

### 4.1 LC stimulation and the global signal

The global signal exhibits an anticorrelated relationship with arousal andvigilance measures across species (Chang et al.,2016;Pisauro et al., 2016;Wong et al., 2013,^2016^). Given this relationship with arousal, we werecurious about how the global signal would be affected by stimulation of the LC.With respect to the spatial distribution of the global signal, LC stimulationdid, indeed, appear to result regionally specific changes. First, the primarylayout of correlation to the global signal that was observed in both the mCherryand ChR2 animals reflected that reported in (Anumba et al., 2023), where the midline structure, which likelyincludes the cingulate cortex, showed the highest correlation to the globalsignal. Interestingly, stimulation of the LC in the ChR2 animals did not equallyincrease correlation to the global signal throughout the brain, but insteadresulted in higher correlation from regions that previously showed elevatedglobal signal correlation (Fig. 3). InAnumba et al. (2023), the DMN wasshown to contribute significantly to the global signal in rats, and whenmatching regions of high global signal correlation to the rat DMN as reported inLu et al. (2012), the stronglycorrelated midline structures comprised the cingulate cortex and parts of theretrosplenial cortex. Studies in humans have also shown that certain DMN nodes,including the posterior cingulate cortex for which the retrosplenial cortex isan ortholog, have shown both higher activity than the global signal (Raichle et al., 2001) and a higheramplitude of ultra-low frequency fluctuations than the global signal (Yu-Feng et al., 2007). The anteriorcingulate cortex (ACC), which corresponds to the cingulate cortex in rodents, isinvolved in a wide array of high-order cognitive functions such as theregulation of emotions, decision making, and learning (Apps et al., 2016;Stevens et al., 2011). A human brain mapping study that consideredbrainstem structures showed that the ACC had the strongest summed functionalconnectivity to all brainstem nuclei, making it a primary hub forcortical-brainstem functional connectivity (Hansen et al., 2023). Stimulation of alpha-2a adrenergic receptorsin non-human primates was reported to increase the encoding of negativeprediction errors in the ACC, linking the noradrenergic system to theACC’s involvement in learning (Hassani& Womelsdorf, 2023). Our lab has also shown the importance ofreciprocal connections between the ACC and LC in affective responses tocontextual novelty (Lustberg, Iannitelli, etal., 2020;Lustberg, Tillage, et al.,2020). Therefore, we asked whether LC stimulation resulted inincreased correlation of the cingulate cortex to the global signal. The largesteffects were observed during tonic 2 Hz and 5 Hz stimulation of the LC, whichare associated with generalized awake states and stress, respectively (Aston-Jones & Bloom, 1981;Aston-Jones & Cohen, 2005;Berridge & Waterhouse, 2003;Carter et al., 2010;McCall et al., 2015;Morris et al., 2020;Prokopiou etal., 2022;Valentino & Foote,1988). Importantly, the increase in correlation of the cingulatecortex to the global signal was apparent within ChR2 animals compared to mCherryanimals as a whole, rather than based on specific stimulation patterns. Onereason for this may be due to the cumulative and lasting effects of LCstimulation over the entire imaging period, which may have influenced globalsignal even during baseline scans. However, we compared metrics of the firstbaseline scan (before stimulation) to the second baseline scan (afterstimulation) in ChR2-expressing animals and found no differences between them(Supplementary Fig.S1). The lack of significant effects on the basis of stimulationpattern indicates that observed trends, such as the increase in cingulate cortexcontribution to the global signal, may be small in nature. It is also importantto note that the group sizes for some stimulation levels, especially for theChR2 animals, were relatively small, making it possible that more power affordedby bigger group sizes could uncover a significant effect.

### 4.2 LC stimulation and QPP2

QPP2 was detected using the robust pattern-detection algorithm reported in (Yousefi et al., 2018), although there wasvery little change in the detection rate as a result of LC stimulation (Fig. 4A). However, the histograms of STCvalues during different levels of stimulation revealed a slightly higher, butnon-significant, percentage of correlation values above the 0.2 threshold in theChR2 animals during 2 Hz and 5 Hz LC stimulation. It is interesting to comparethese findings with a preliminary study in which either elevating extracellularNE levels with the NE transporter inhibitor atomoxetine or depleting NE usingthe LC-specific neurotoxin DSP4 disrupted the generation of QPPs (Abbas et al., 2018). By contrast, in the currentstudy we found that tonic stimulation of the LC*increases*thedetection of QPPs. An important distinction between these two studies is thatatomoxetine and DSP4 induce dramatic changes in LC-NE signaling, whereasoptogenetic stimulation triggers more subtle effects that mimic physiologicalfunction. One interpretation consistent with the “adaptive gaintheory” and inverted U dose-response nature of LC function (Aston-Jones & Cohen, 2005) is that“optimal” LC-NE transmission promotes QPP2, while pathologicalchanges to LC circuits associated with neuropsychiatric and neurodegenerativediseases intrude on these brain states (Iannitelli & Weinshenker, 2023;M. Kelberman et al., 2020;Poe et al., 2020;Weinshenker, 2018;Weinshenker& Holmes, 2016).

The relative regions involved in the detected QPP as well as their activityduring the pattern were also observed. The foundational structure of the QPP wasvery similar across groups and stimulation levels, with certain regionsexhibiting stronger activity within the pattern and those regions belonging toone of two groups of structures whose activity is anticorrelated (Fig. 4B-C). In particular, the somatosensory,insular, and striatal networks move in phase with each other whereas theretrosplenial cortex, hippocampus, visual network, and a few midbrain structureswere in phase with each other. These groupings of regions within the QPP largelyreflect what has previously been reported for QPPs in rats (Majeed et al., 2011;van den Berg et al., 2022). Interestingly, both of these studiesshow strong involvement of the cingulate cortex in the QPP at rest, whereas inour study strong activity from the cingulate cortex is only observed at thegroup level during stimulation of the LC at 5 Hz tonic and 15 Hz phasic levels.Under these conditions, the activity of the cingulate cortex is in phase withthe retrosplenial cortex and hippocampus, both of which, in addition to thecingulate cortex, have been defined as part of the rat DMN (Lu et al., 2012). LC firing at 5 Hz tonic and 15 Hzphasic levels is associated with anxiety and focused attention, respectively(Berridge & Waterhouse, 2003;Carter et al., 2010;McCall et al., 2015). These results indicate that LCactivity during states of high arousal, regardless of valence, could lead to anincrease in activity of the cingulate cortex in rats, especially in the contextof slow spatiotemporal patterns like QPPs. Importantly, the effects ofstimulation on increases in cingulate activity in the QPP were not statisticallysignificant, indicating that this effect also may be small in nature. That beingsaid, it known that the pattern-detecting algorithm used to detect the QPPs ismore robust at the group level (i.e., with more data) and that results on theindividual level are not necessarily indicative of the group-level results(H. Watters et al., 2024). It shouldalso be noted that during 5 Hz LC stimulation there was a slight phase shift inthe activity of the insular and striatal networks, meaning that their peaks inactivity occurred slightly before those of the other structures. Though thiseffect has not previously been reported, it is a point of interest that could befurther investigated.

### 4.3 CPCA findings

We used CPCA to assess whether LC stimulation had any effect on the presentationand occurrence of the first three principal components (QPP1, QPP2, QPP3) inthese rats. A recent study showed that the majority of large-scalespatiotemporal signals that have been detected with fMRI can be explained by thefirst three principal components, or QPPs, of activity as calculated throughCPCA (Bolt et al., 2022). Specifically,they showed that QPP1 was reflective of the global signal and that QPP2 wasindicative of what is defined as the anticorrelated QPP. Moreover, recent workin rats showed that different anesthetic conditions alter the ratio of QPPs, andin humans, QPP1 increases at the expense of QPP2 over the course of a scan(Meyer-Baese et al., 2024b).

LC stimulation appeared to have very subtle effects on the incidence of theprincipal components, despite consistent trends being observed based on whichvirus animals expressed (Fig. 5). ChR2expression should not cause significant changes alone, and we believe thiseffect could be due to lasting effects of LC stimulation across scans inChR2-expressing animals or as a result of other variables, such as anesthesia,which can produce large changes in the distribution of these QPPs (Meyer-Baese et al., 2024b). Regardless, thecomparison that produced the largest effect size was the incidence of QPP2during 5 Hz stimulation (approximately 10% lower occurrence in ChR2 animals).This is worth noting because our other findings using the QPP pattern-detectingalgorithm reveal that stimulation at 5 Hz induced changes in the presentation ofthe QPP (Fig. 4). Given this and the factthat QPP2 is reflective of the anticorrelation between DMN and TPN (Bolt et al., 2022), it is possible thatthese changes during 5 Hz stimulation could be responsible either for the signalthat was detected as QPP2 when using CPCA or for the reduced frequency of QPP2occurrence that we see in these scans.

### 4.4 Potential subtle effects

Despite the LC having global neuromodulatory influence, the majority of theresults presented here display effects that are quite subtle, highlighted by thelack of statistical significance. Moreover, observed effects were restricted tospecific brain regions or structures as opposed to the whole brain. It should benoted that the effects of stimulating small nuclei using optogenetic-fMRI areusually quite small. Studies using optogenetics to stimulate the ventralposteromedial nucleus (VPM) of the thalamus and LC have reported significant yetsmall changes in % BOLD signal as a result of optogenetic stimulation (Chuang et al., 2023;Grimm et al., 2022). In contrast, our study did notshow significant effects as a result of LC stimulation, and the effects seenwere not observed on a global scale as has been reported by others (Oyarzabal et al., 2021;Zerbi et al., 2019). However, the latter may beattributed to the differences in method of LC stimulation. Correspondingly, thesensitivity of optogenetic-fMRI is thought to potentially underreportwhole-brain responses when used to stimulate small areas (Lee et al., 2022).

As mentioned above, the small effects that were observed could also be explainedby the Yerkes-Dodson model in which the relationship between arousal andperformance is explained by an inverted-U-shaped curve, with a small range ofarousal leading to optimal performance (P. A.Watters et al., 1997). This inverted-U curve has also been shown toexplain the relationship between LC-NE modulation and network function andbehavior (Aston-Jones & Cohen,2005;Poe et al., 2020). Whencompared to extreme forms of LC-NE manipulation (Abbas et al., 2018), optogenetic modulation of the LC allows a morefine-tuned approach over a narrow range of activity that may result in moresubtle effects. Additionally, although our study was designed to account forlittle interference of between-scan effects, a study showed that high tonicfiring of the LC can reduce the excitability and NE release of subsequent phasicfiring modes (Li et al., 2023).Therefore, it is possible that the length of our scans (10 minutes) withconstant stimulation may have resulted in overall NE*depletion*,resulting in a lack of effects observed over the whole length of the scan.Reanalysis using a shorter stimulation period would be a necessary direction forfuture study. Given the nature of the BOLD signal and its dependence on signalfrom the brain vasculature, it is important to recognize the role of NE as avasoconstrictor and how this could have resulted in competing effects to thesignal captured during LC stimulation (Bekar etal., 2012). This also speaks to the complexity of the neural andhemodynamic systems involved when measuring these spatiotemporal signals withfMRI. Lastly, despite the valuable advancements that work in rodents allows(Pagani et al., 2023), it isimportant to note that the animals presented were scanned under anesthesia, astate which is known to affect measures of brain activity (X. Liu et al., 2013;Masamoto et al., 2009;Masamoto& Kanno, 2012), and may compete with arousal-associatedmanipulations such as certain levels of LC stimulation.

### 4.5 Localized effects of LC stimulation

The most consistent and perhaps interesting finding of the current study is thatLC stimulation results in localized effects on brain-wide dynamics, most notablythe cingulate cortex. One explanation for this effect could be our viral andoptic fiber targeting, which delivered virus and implanted fibers proximal anddorsal to the LC, respectively. This could result in variable viral expressionand stimulation, therefore engaging different efferent projection targets acrossanimals. This is somewhat unlikely given the observation of mCherry expressionand widespread activation of neurons throughout the LC (Fig. 2C&D). Inaddition, we would expect any effects to be washed out by variable targetingacross animals, instead of the stark localized effects we observed. Nonetheless,a comprehensive analysis of viral expression and c-Fos throughout the LC axes onan individual animal basis would be required to ascertain the true effect ofvariability on global brain dynamics but is outside the scope of the currentstudy.

Instead, we posit that localized effects of LC stimulation are due to modularityin the form and function of the LC. The LC has been traditionally viewed as anevolutionarily conserved, homogenous nucleus, defined by nearly universal longbranching axons throughout the brain that release NE via volumetransmission/non-synaptic release (Atzori etal., 2016;Ehrenberg et al.,2023;Mather et al., 2016;Vreven et al., 2024;Wang et al., 2022). However, recent findings haveadded nuance to this view. For example, single LC neurons exhibit largelynon-overlapping projections based on physical location within the LC core (D. J. Chandler et al., 2014,^2019^;D.Chandler & Waterhouse, 2012;Loughlin et al., 1986;Poe et al.,2020;Schwarz & Luo,2015;Sulkes Cuevas et al.,2023). These efferent projections dictate electrophysiologicalproperties and biochemical makeup of individual LC neurons (D. J. Chandler et al., 2014;Schwarz & Luo, 2015). Discrete behaviorsinfluenced by LC-NE signaling, such as aversion and anxiety, appear more likelyto engage LC modules that project to regions promoting such behaviors (in thecase of aversion/anxiety, the amygdala, and prefrontal and cingulate cortices)(Hirschberg et al., n.d.;Poe et al., 2020;Uematsu et al., 2017). Notably, the LC-cingulatecircuit is critical for a range of responses, including arousal, pupil dilation,stress, and attention (Chintalacheruvu et al.,2024;Hallock et al., 2024;Joshi & Gold, 2022;J. Liu et al., 2024;Lustberg, Tillage, et al., 2020, p. 202), all ofwhich are likely to be elicited by our various stimulation parameters.

Apart from the potential impacts of heterogenous LC organization on global braindynamics, response to LC stimulation in target regions is influenced by theexpression profile of NE receptors. Two previous studies demonstrated strongmodulation of cingulate cortex functional connectivity in response to LCchemogenetic stimulation (Oyarzabal et al.,2021;Zerbi et al., 2019).Furthermore, strong positive correlations between functional connectivity and NEreceptor expression were found in response to chemogenetic LC stimulation (Zerbi et al., 2019). This effect was trueof the cingulate cortex, which expresses some of the highest levels ofadrenergic receptors. Thus, the emerging appreciation for modular LC circuitsand receptor distribution profiles could, at least in part, account for thelocalized effects observed in our study.

### 4.6 Limitations and future directions

One limitation of this study is the unilateral nature of our LC optogeneticstimulation. Plenty of studies have induced behavioral phenotypes utilizingunilateral optogenetic LC stimulation (Marzo etal., 2014;McCall et al.,2015;Tillage et al., 2020,^2021^). However, a majority of LC fibersproject ipsilaterally and some have produced unilateral effects as a result ofLC stimulation (Grimm et al., 2022;Marzo et al., 2014;Proudfit & Clark, 1991;Waterhouse et al., 1983,^2022^), something that this study was unable to assessdue to the lack of bilateral symmetry in the functional atlas used duringanalysis. Therefore, more robust effects may be observed with bilateralstimulation, as has been observed previously (Zerbi et al., 2019). Furthermore, while we utilized optogenetics forthe added benefits of controlling precise timing and patterns of LC stimulation,this approach is limited by light penetration through the tissue, variable viralexpression, and placement of optic ferrules. All of these could impact the totalnumber of LC neurons and location within the LC core that were responsive tooptogenetic stimulation, as noted earlier. Follow-up studies could isolate localeffects of LC activity on specific brain regions by stimulating LC axonterminals. Lastly, the reported activity of certain brainstem structures shouldbe taken cautiously due to the use of a surface coil which was unable to obtainoptimal signal from structures at this depth.

## 5 Conclusion

In this study, we combined optogenetic stimulation of the LC with whole-brain fMRIrecordings to assess the effects of LC activity on large-scale spatiotemporaldynamics. Specifically, we assessed the spatial components of the BOLD globalsignal, the presentation of QPPs, and the principal components extracted from CPCAas a result of LC stimulation. Our findings provide evidence that LC stimulation atspecific levels results in small regionally specific effects with regards to theglobal signal and QPPs. LC stimulation showed little change in the frequency ofoccurrence of CPCA principal components. These results suggest that the effects ofLC stimulation are not simultaneously widespread and large in scale, but insteadresult in more regional effects that are small in nature. This study contributes anadditional insight to our wholistic understanding of how resting-state globaldynamics are affected by ongoing neuromodulation and differing brain states such asarousal.

## Supplementary Material

Supplementary Material

## Data Availability

A MATLAB script with the code necessary to perform the reported analyses will be madeavailable upon request and distributed via GitHub. rs-fMRI data will be availableupon request.
